# Need for and Access to Health Care and Medicines: Are There Gender Inequities?

**DOI:** 10.1371/journal.pone.0057228

**Published:** 2013-03-07

**Authors:** Anita K. Wagner, Amy J. Graves, Zhengyu Fan, Saul Walker, Fang Zhang, Dennis Ross-Degnan

**Affiliations:** 1 Department of Population Medicine, Harvard Medical School and Harvard Pilgrim Health Care Institute, Boston, Massachusetts, United States of America; 2 UK Department for International Development, Maputo, Mozambique; Sapienza University of Rome, Italy

## Abstract

**Objective:**

Differences between women and men in political and economic empowerment, education, and health risks are well-documented. Similar gender inequities in access to care and medicines have been hypothesized but evidence is lacking.

**Methods:**

We analyzed 2002 World Health Survey data for 257,922 adult respondents and 80,932 children less than 5 years old from 53 mostly low and middle-income countries. We constructed indicators of need for, access to, and perceptions of care, and we described the number of countries with equal and statistically different proportions of women and men for each indicator. Using multivariate logistic regression models, we estimated effects of gender on our study outcomes, overall and by household poverty.

**Findings:**

Women reported significantly more need for care for three of six chronic conditions surveyed, and they were more likely to have at least one of the conditions (OR 1.41 [95% CI 1.38, 1.44]). Among those with reported need for care, there were no consistent differences in access to care between women and men overall (e.g., treatment for all reported chronic conditions, OR 1.00 [0.96, 1.04]) or by household poverty. Of concern, access to care for chronic conditions was distressingly low among both men and women in many countries, as was access to preventive services among boys and girls less than 5 years old.

**Conclusions:**

These cross-country results do not suggest a systematic disadvantage of women in access to curative care and medicines for treating selected chronic conditions or acute symptoms, or to preventive services among boys and girls.

## Background

Gender differences have become a key global concern. Governments [1]–[3], the United Nation [4], and the World Health Organization (WHO) [5] have established gender-focused development programs which generally refer to gender equality, i.e., equal representation of women and men, in government bodies, leadership positions, and at educational levels. The WHO has called for inclusion of a gender perspective in national essential medicines programs [6]. Such a perspective implies moving towards gender equity, that is, equal access to and use of medicines for women and men who have similar needs, which vary by gender and throughout the life course.

Increased risk of death among women from child birth, unsafe abortion, gender-related violence, and sexually transmitted diseases including HIV infection [7] and limited progress toward achieving the gender-focused Millennium Development Goal (MDG) 4, to reduce child mortality, and MDG 5, to improve maternal health [8], are well documented. While researchers have begun to focus on gender differences in care [9]–[14], almost no empirical data exist to inform policy makers on the role of gender in need for and access to medicines or on useful targets for gender equity initiatives [15].

This research uses large-scale survey data to address the following questions: Are there systematic gender differences in need for and access to treatment for chronic and acute conditions among adults; or in access to preventive or curative care among children under 5 years old?

## Methods

### Ethics statement

The study was determined to be exempt from human subjects review by the Human Studies Committee of Harvard Pilgrim Health Care.

### Data sources and measures

We used data from the World Health Survey (WHS) [16], [17] conducted by the WHO in 2002 and 2003 in 70 countries. Detailed information on household and respondent selection, as well as data quality results are available elsewhere [18], [19]. Briefly, using multi-stage cluster randomized sampling, country household samples were drawn from nationally representative sample frames. In the 53 study countries, household respondents, selected from all eligible adult household members based on Kish tables [18], completed the long version of the survey in face-to-face interviews with trained interviewers. Country samples consisted of 668 to 38,614 households and 585 to 38,618 individuals, with high household (median 89%; range 24%–100%) and individual (median 98%, range 63%–100%) response rates across countries [20].

The adult respondent answered mostly closed-ended questions about health problems, care seeking, and care received for themselves and for the youngest child under 5 years old in the household; they also provided information about household characteristics including assets, expenditures, and insurance coverage of members. We have previously reported on household access to care, burden of health care expenditures, and household risk protection [21].

For the present analysis, we constructed indicators of need for care; access to care and medicines; and perceptions of the health care system.

### Need for care

To describe *adults' need for health care and medicines*, we assessed reports of moderate, bad, or very bad health and moderate, severe, or extreme limitations in daily activities; having ever been diagnosed with or in the past 12 months experienced symptoms of one or more of six chronic conditions (arthritis, angina, asthma, depression, schizophrenia, and diabetes); and needing care in the past year for acute symptoms (high fever, severe diarrhoea, cough). The target chronic conditions included in the WHS were selected due to a high global burden of disease and were assessed using questions from established survey tools, where available [19], [22].

We defined *children's need for care* as the youngest child less than 5 years in the household having ever experienced fever, diarrhoea, or another illness.

### Access to care and medicines

For *adult respondents*, we defined *access to care and medicines for a chronic condition* as having received medications or other treatments during the past two weeks for the six target chronic conditions. For those who reported need for acute care in the past year, we defined overall *access to acute care* as receiving care when last needed for high fever, severe diarrhoea, or cough and *access to medicines* as getting all or most medicines prescribed during the last health care encounter for conditions generally treatable with medicines (high fever, severe diarrhoea, cough, arthritis, asthma, heart disease).

For *children under 5 years*, we defined *access to care* according to specific measures in the WHS, namely having received: at least one Vitamin A capsule in the past 12 months; one measles and at least one diphtheria, tetanus, or whooping cough (DPT) vaccination; all three DPT vaccinations; or care for fever, diarrhoea, or any other illness when last needed. We also defined *access to malaria treatment* in children as having received any treatment for malaria and an antimalarial prescribed by a medical professional during the last episode of fever.

### Perceptions of care

We defined several measures of respondents' *perceptions of care*: one indicator of satisfaction with “the way health care runs” in their country and indicators of perceived discrimination based on feelings of having been treated worse by outpatient health care providers because of gender, type of illness, social class, or lack of money.

### Household and respondent characteristics

We characterized households and respondents according to socioeconomic and demographic characteristics, including: household size greater than or equal to 6 members; having a household member age 60 years and older and/or a child under 5 years; highest education of any household member as none or less than primary school; household wealth in the lowest two income quintiles based on household assets (described as “poor”); urban location; coverage of household members (all, some, none) by mandatory or voluntary health insurance; and respondent age (18–29 years, 30–59 years, 60+ years), marital status, and education (either no formal schooling or less than primary school).

### Analysis

The WHS employed a complex survey design with differing weights, stratification, and clustering in each country. Our descriptive within-country analyses adjust for the survey design where possible, using sample weights provided with the data. We describe results for men and women respondents and for girls and boys, both for all households and separately for poor households.

We used multi-country individual-level logistic regression models (without country-level survey sampling weights) to explore the relationships between gender and need for care, access to care and medicines, and perceptions of care. To assess whether gender effects differed by household poverty, we included gender and poverty interaction terms in the models and estimated gender effects among those in the lowest two income quintiles (“poor”) versus the other three income quintiles (“non-poor”). We also used robust variance estimation [23] to estimate gender effects when logistic main effects models suggested gender differences, to account for possible within-country correlations. Since results were similar, we only report logistic regression estimates.

In all multivariate analyses, estimates of gender differences are adjusted for the household and respondent characteristics listed above. Since we selected household and respondent characteristics on conceptual grounds, we did not apply variable elimination strategies. We used SAS version 9.1 for the analyses and p<0.05 as the significance threshold. Study data were provided in de-identified country datasets by the WHO.

## Results

We analyzed data from 257,922 adult respondents and 80,932 children less than 5 years old from 53 mostly low and middle income countries [24] in East Asia and Pacific (n = 6 countries; 3 low [LIC], 2 lower-middle [LMIC], and 1 upper-middle [UMIC] income); Europe and Central Asia (n = 14; 6 high-income countries [HIC], 3 LMIC, 5 UMIC); Latin America and the Caribbean (n = 7; 4 LMIC, 3 UMIC); Middle East and North Africa (n = 3; 1 HIC, 2 LMIC); South Asia (n = 5; 3 LIC, 2 LMIC); and Sub-Saharan Africa (n = 18; 13 LIC, 3 LMIC, 2 UMIC). Country-level results are listed in Tables S1, S2, S3, S4, S5, S6, S7, S8.

### Respondent demographics

**Figure 1:**
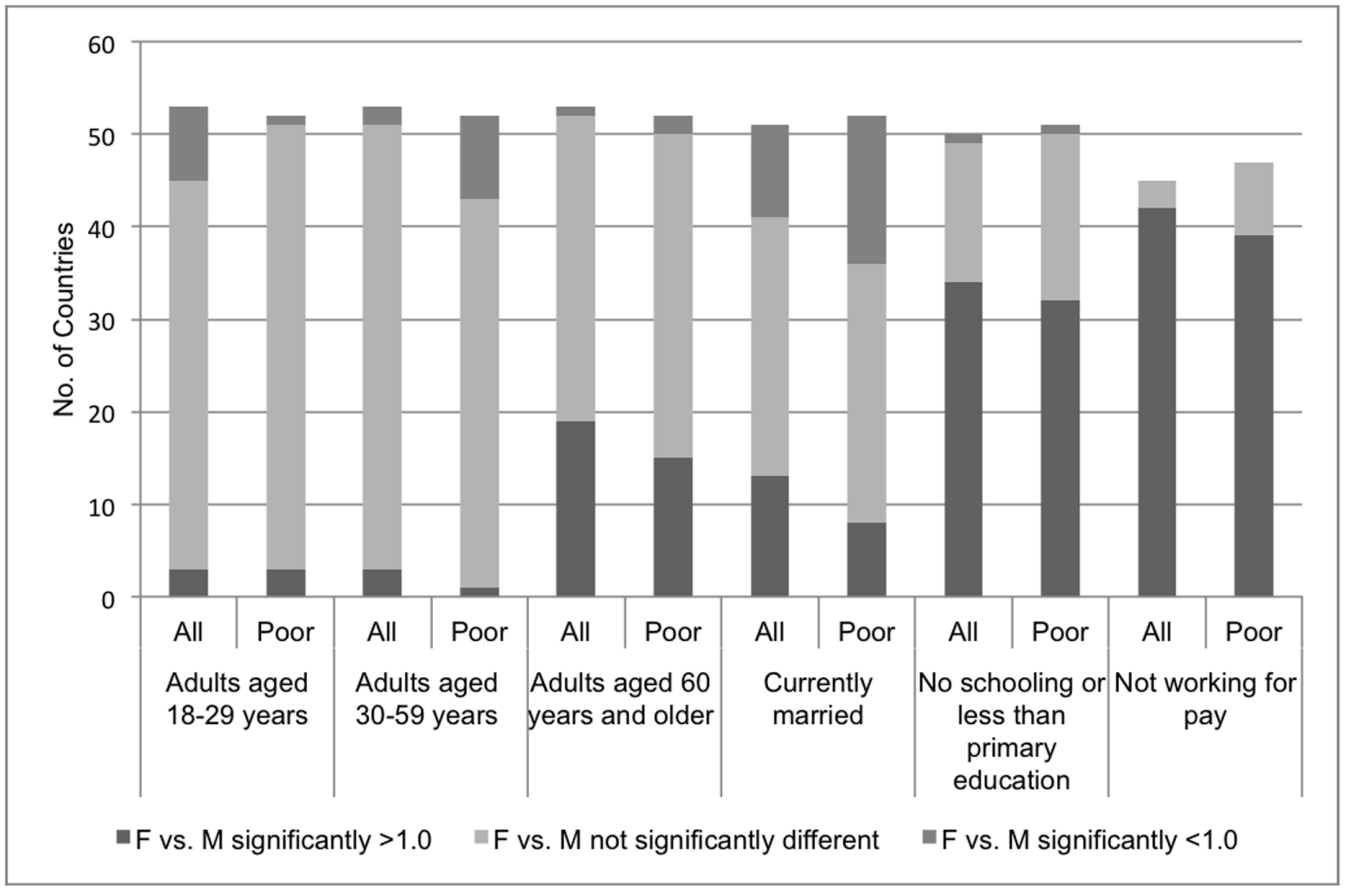
Adult demographics, number of countries with significant gender differences, all versus poor households.

Table 1 describes respondent characteristics across countries. The median proportions of women age 60 and older, with limited schooling, and not working for pay were significantly higher than the corresponding proportions of men. Figure 1 summarizes these country-level data by displaying for each characteristic three groups: the number of countries in which the percentage of women with the characteristic was significantly greater than the percentage of men; the number in which these percentages were not significantly different; and the number of countries where the percentage of men was significantly greater. In substantial numbers of countries, significantly more women were older (n = 19 countries), less educated (n = 34), and not working for pay (n = 42). Since age, education, and employment could confound analyses of gender differences in need for and access to care, we adjusted for differences in these respondent demographic characteristics in multivariate analyses.

**Table 1:** Median (interquartile range) of percentage of characteristics of adult respondents and their households in 53 countries.

	Respondents from all households		Respondents from poor households	
	Female, n = 143238	Male, n = 114684	Female, n = 53155	Male, n = 41810
Number of adults	2453 (1534, 2828)	1830 (1165, 2354)	860 (517, 1107)	677 (433, 919)
Age 18–29 years	35.3 (24.6, 39.9)	37.9 (26.1, 42.4)	35.6 (23.1, 39.2)	36.4 (22.2, 39.8)
Age 30–59 years	50.2 (48.0, 51.9)	50.8 (47.0, 53.4)	48.8 (42.5, 51.0)	49.7 (45.3, 52.4)
Age≥60 years	12.4 (10.8, 20.7)	11.0 (9.3, 19.4)	13.7 (11.3, 24.4)	12.4 (9.8, 25.8)
Currently married	59.1 (50.1, 68.0)	60.6 (52.2, 69.9)	57.9 (46.1, 68.6)	61.6 (49.5, 71.0)
No schooling or less than primary education	29.7 (12.6, 63.8)	24.0 (10.2, 47.8)	45.7 (20.2, 83.8)	35.7 (21.0, 69.9)
Not working for pay	62.7 (49.6, 68.7)	28.4 (18.3, 39.6)	68.3 (56.5, 76.0)	29.4 (13.9, 52.7)

### Need for care among adults

More than one-third of respondents rated their health as moderate, bad, or very bad. The percentage of individuals reporting chronic conditions varied across countries, but was generally small (Tables S1, S2, S3, S4, S5, S6).

**Figure 2:**
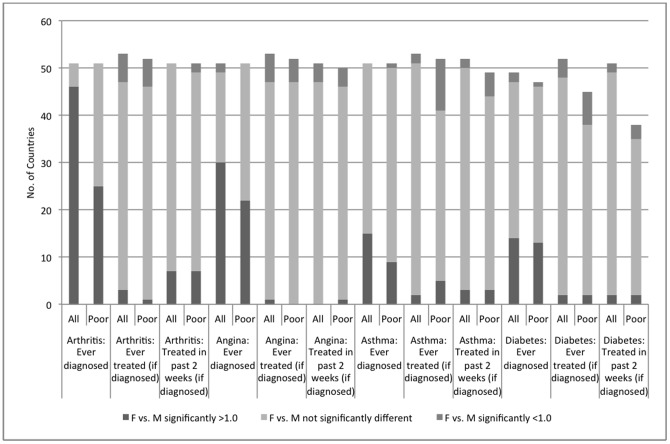
Chronic conditions among adults, number of countries with significant gender differences, all versus poor households. Chronic conditions (arthritis, angina, asthma, diabetes) among adults, number of countries with significant gender differences, all versus poor households.

Across countries, larger proportions of women reported need for care than men (Table 2). In more than 80% of countries, significantly greater percentages of women reported lower health status and greater limitations in daily activities than men (Figures 2-1 and 2-2). Among the poor, differences between men and women were fewer, but still significant in more than half of the countries.

**Table 2:** Median (interquartile range) of percentage of adult respondents with need for and access to care in 53 countries.

	Respondents from all households		Respondents from poor households	
	Female, n = 143238	Male, n = 114684	Female, n = 53155	Male, n = 41810
Number of adults per country	2453 (1534, 2828)	1830 (1165, 2354)	860 (517, 1107)	677 (433, 919)
**Need for care**				
Self-rated health moderate, bad, very bad	45.0 (31.7, 49.6)	33.9 (22.4, 39.6)	47.2 (36.4, 61.8)	39.5 (27.8, 47.4)
Moderate, severe or extreme difficulty with work or household activities	27.4 (19.3, 33.8)	20.2 (14.1, 26.7)	33.1 (22.4, 41.0)	25.5 (16.8, 34.6)
Arthritis: ever diagnosed	15.2 (10.3, 20.4)	9.7 (5.6, 13.7)	17.2 (11.1,23.1)	10.7 (6.7, 16.8)
Angina: ever diagnosed	6.8 (4.9, 11.6)	5.3 (3.1, 8.1)	8.1 (4.7, 13.7)	6.4 (3.3, 10.4)
Asthma: ever diagnosed	4.7 (3.4, 6.5)	3.9 (2.7, 5.2)	5.3 (3.5, 6.6)	3.7 (2.4, 6.1)
Depression: ever diagnosed	5.0 (2.2, 9.2)	2.9 (1.4, 4.6)	4.8 (2.0, 9.8)	2.6 (1.4, 5.0)
Schizophrenia: ever diagnosed	0.8 (0.4, 1.5)	0.8 (0.4, 1.3)	0.9 (0.5, 1.8)	1.0 (0.6, 1.7)
Diabetes: ever diagnosed	2.9 (1.1, 5.5)	2.2 (1.1, 3.8)	2.5 (0.8, 5.0)	1.4 (0.6, 2.6)
**Access to care among those with need for care**				
Arthritis treatment in last 2 weeks	42.6 (28.9, 49.9)	34.9 (27.0, 44.9)	39.0 (24.1, 51.3)	32.9 (21.5, 45.9)
Angina treatment in last 2 weeks	37.6 (26.4, 62.4)	46.3 (29.2, 60.4)	33.1 (22.8, 64.5)	38.3 (21.9, 61.0)
Asthma treatment in last 2 weeks	42.6 (34.5, 54.8)	41.1 (30.2, 54.4)	38.9 (31.9, 57.5)	44.4 (23.0, 51.9)
Depression treatment in last 2 weeks	30.5 (23.4, 38.0)	27.9 (17.2, 43.7)	29.6 (15.1, 42.0)	28.1 (13.5, 44.9)
Schizophrenia treatment in last 2 weeks	34.7 (22.5, 57.2)	40.8 (27.6, 51.9)	33.2 (14.9, 61.4)	35.6 (21.9, 60.6)
Diabetes treatment in last 2 weeks	53.9 (37.8, 64.0)	54.6 (42.8, 69.3)	48.2 (25.8, 62.2)	47.8 (23.4, 72.9)
Received acute care when needed in past year	96.4 (93.1, 98.6)	95.9 (92.6, 99.2)	95.9 (87.3, 99.5)	94.1 (88.6, 99.1)
Received all or most medicines prescribed during last visit	75.7 (67.5, 84.0)	77.8 (65.8, 82.5)	71.4 (63.1, 80.9)	72.6 (62.5, 83.4)

**Figure 3:**
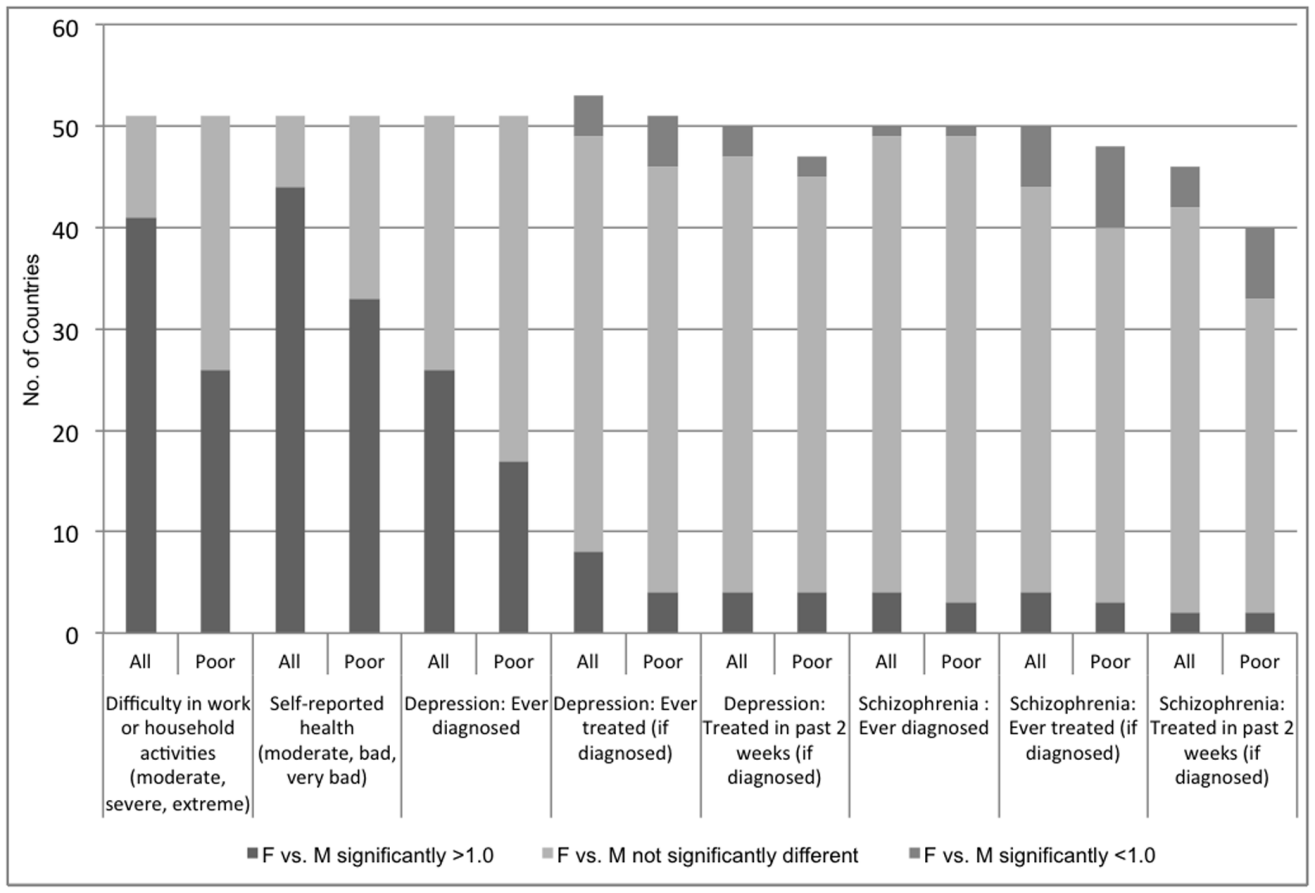
Chronic conditions among adults, number of countries with significant gender differences, all versus poor households. Chronic conditions among adults (activity limitations, self-reported health, depression, schizophrenia), number of countries with significant gender differences, all versus poor households.

In more than half of the countries, significantly greater percentages of women reported diagnoses of arthritis, angina (Figure 2), and depression (Figure 3); in about 20% of countries, significantly greater percentages of women reported diagnoses of asthma and diabetes (Figure 2). Among the poor, fewer countries exhibited significant gender differences.

In multivariate analyses (Table 3), women were significantly more likely than men to rate their health as poor (odds ratio 1.36, 95% confidence interval [1.33,1.38]), to report difficulties with activities (1.21 [1.18,1.24]), to need care for acute symptoms (1.45 [1.39, 1.52]) and to have been diagnosed with or experienced symptoms of at least one of six chronic conditions (1.41 [1.38,1.44]), arthritis (1.46 [1.43,1.50]), angina (1.37 [1.34,1.41]), and depression (1.56 [1.51,1.60]).

**Table 3:** Median (interquartile range) of percentage of children under 5 years old with access to prevention, need for and access to curative care in 53 countries.

	Children from all households		Children from poor households	
	Female, n = 39640	Male, n = 41292	Female, n = 16506	Male, n = 17152
Number of children	601 (202, 948)	651 (202, 999)	256 (72, 407)	265 (74, 400)
At least one Vitamin A capsule in past 12 months	57.1 (30.6, 76.3)	55.3 (30.7, 73.3)	52.9 (26.0, 75.0)	55.1 (27.9, 69.2)
At least measles and one DPT vaccine received	39.2 (27.0, 58.4)	39.6 (26.8, 58.7)	36.8 (21.9, 58.5)	40.4 (26.1, 58.5)
Fever, diarrhoea, or other illness within last month	37.7 (27.8, 45.4)	38.6 (29.1, 45.9)	37.7 (24.3, 44.3)	39.0 (31.3, 47.5)
Care received for last illness	87.4 (80.2, 91.8)	85.8 (79.2, 90.7)	83.7 (73.9, 90.7)	82.2 (75.2, 88.3)
Care received in hospital	24.8 (16.3, 41.5)	27.5 (16.6, 43.4)	24.3 (14.3, 37.2)	33.3 (20.1, 49.5)
Care received in public facility	85.9 (75.7, 93.6)	86.5 (71.1, 93.3)	90.5 (79.6, 96.2)	92.1 (80.5, 98.7)
Treatment for malaria during last episode of fever	47.7 (9.2, 79.1)	48.8 (11.0, 77.9)	46.1 (11.3, 75.4)	45.3 (11.5, 77.5)
Anti-malarial prescribed by professional for fever treated for malaria	98.2 (90.3, 100.0)	97.5 (93.5, 100.0)	98.9 (87.7, 100.0)	97.1 (86.5, 100.0)

### Access to care among adults

**Figure 4:**
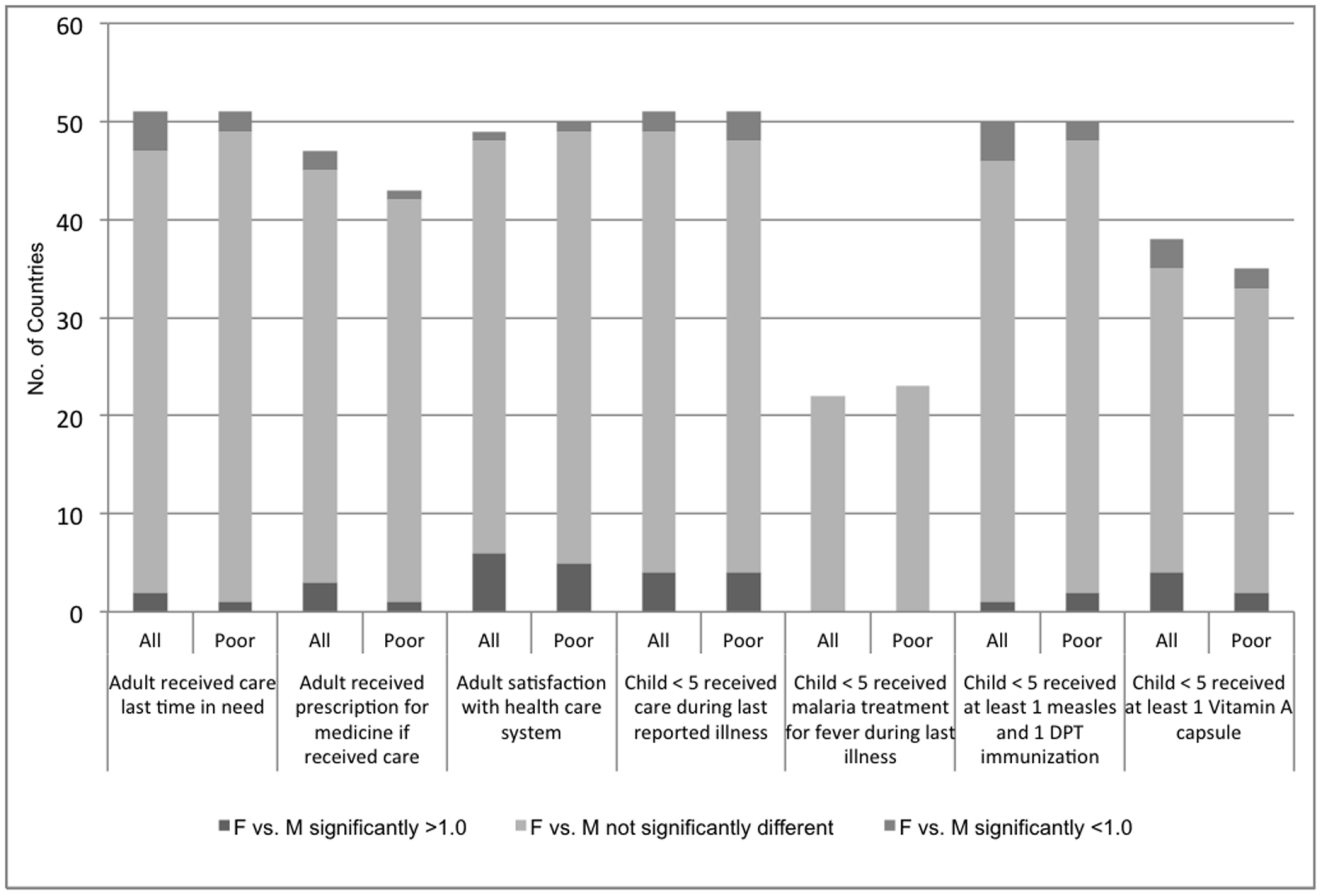
Access to care, number of countries with significant gender differences, all versus poor households.

Across countries, generally less than half of adults diagnosed with a chronic condition reported receiving medication or other treatments in the past 2 weeks (Table 2). Women and men with chronic conditions reported similarly low rates of access to medications and other treatments, both overall and among the poor (Figures 2-1 and 2-2). In contrast, men and women both reported very high rates of access to acute care when last needed (greater than 95%), overall and among the poor (Table 2). In most countries and for the poor, access to care when last needed and access to prescribed medicines during the last health care encounter did not differ significantly between women and men (Figure 4).

In multivariate analyses, women with arthritis, asthma, and depression reported receiving medication treatment for these conditions significantly more frequently than men. The genders did not differ significantly in access to medications for all chronic conditions measured, or for angina, schizophrenia, and diabetes; in access to acute care when last needed; or in access to prescribed medicines during the last encounter (Table 4).

**Table 4:** Relationship of gender to need for, access to care among adult respondents*.

	Odds ratio, all households (95% confidence interval)	Odds ratio, poor households (95% confidence interval)	Odds ratio, less poor households (95% confidence interval)
**Need for care**			
Self-rated health moderate, bad, very bad	1.36 (1.33, 1.38)	1.37 (1.33, 1.41)	1.35 (1.31, 1.38)
Moderate, severe or extreme difficulty with work or household activities	1.21 (1.18, 1.24)	1.14 (1.10, 1.18)#	1.27 (1.23, 1.31)#
At least one chronic condition	1.41 (1.38, 1.44)	1.40 (1.36, 1.44)	1.42 (1.38, 1.45)
Arthritis diagnosis or symptoms	1.46 (1.43, 1.50)	1.42 (1.37, 1.47)	1.49 (1.44, 1.54)
Angina diagnosis or symptoms	1.37 (1.34, 1.41)	1.38 (1.33, 1.43)	1.37 (1.32, 1.42)
Asthma diagnosis or symptoms	1.01 (0.98, 1.04)	0.98 (0.94, 1.03)	1.03 (0.99, 1.07)
Depression diagnosis or symptoms	1.56 (1.51, 1.60)	1.48 (1.41, 1.55)#	1.62 (1.55, 1.68)#
Schizophrenia diagnosis	1.01 (0.92, 1.09)	1.06 (0.93, 1.20)	0.96 (0.86, 1.08)
Diabetes diagnosis	1.05 (1.00, 1.11)	1.25 (1.14, 1.36)#	0.96 (0.90, 1.02)#
Needed acute care within past year	1.45 (1.39, 1.52)	1.42 (1.32, 1.53)	1.47 (1.39, 1.56)
**Access to care and medicines**			
Treatment for all reported chronic conditions	1.00 (0.96, 1.04)	1.08 (1.01, 1.15)#	0.95 (0.90, 1.00)#
Arthritis treatment	1.22 (1.16, 1.28)	1.27 (1.18, 1.38)	1.18 (1.10, 1.26)
Angina treatment	0.95 (0.90, 1.02)	1.04 (0.94, 1.14)#	0.90 (0.82, 0.97)#
Asthma treatment	1.19 (1.11, 1.28)	1.21 (1.09, 1.35)	1.17 (1.07, 1.29)
Depression treatment	1.18 (1.08, 1.29)	1.24 (1.08, 1.43)	1.14 (1.02, 1.28)
Schizophrenia treatment	0.94 (0.78, 1.13)	0.93 (0.70, 1.23)	0.95 (0.73, 1.23)
Diabetes treatment	1.01 (0.91, 1.12)	1.11 (0.93, 1.32)	0.96 (0.85, 1.09)
Acute care when needed in past year	0.95 (0.85, 1.06)	0.86 (0.73, 1.00)	1.04 (0.90, 1.21)
All or most medicines needed during last visit	0.98 (0.93, 1.03)	0.97 (0.89, 1.05)	0.99 (0.92, 1.06)

* Females are coded as 1 in the models. Models control for household size; having a member age 60 years and older or a child under 5 years (adult models only); highest education of any household member; household poverty; urban location; insurance coverage; respondent age, marital status, education, and health status. Access to care models are for populations with need for care.

# Indicates that odds ratios among poor and non-poor differ significantly (p<0.05).

Of interest, women were significantly more likely to report being satisfied with their country's health care system than men. There were no gender differences in perceptions of discrimination in outpatient care due to gender; but women were significantly less likely than men to report perceived discrimination due to type of illness, social class, or lack of money (Table 5).

**Table 5:** Relationship of gender to perceptions of care among adult respondents*.

	Odds ratio, all households (95% confidence interval)	Odds ratio, poor households (95% confidence interval)	Odds ratio, less poor households (95% confidence interval)
**Perceptions of care**			
High satisfaction with health care in country	1.15 (1.13, 1.17)	1.14 (1.11, 1.17)	1.15 (1.13, 1.18)
Perceived discrimination in outpatient care due to gender	1.00 (0.88, 1.14)	1.12 (0.90, 1.40)	0.94 (0.80, 1.11)
Perceived discrimination in outpatient care due to illness	0.85 (0.75, 0.96)	0.80 (0.66, 0.98)	0.87 (0.75, 1.02)
Perceived discrimination in outpatient care due to social class	0.88 (0.81, 0.94)	0.82 (0.73, 0.91)	0.92 (0.84, 1.01)
Perceived discrimination in outpatient care due to lack of money	0.88 (0.83, 0.93)	0.85 (0.78, 0.93)	0.90 (0.84, 0.97)

* Females are coded as 1 in the models. Models control for household size; having a member age 60 years and older or a child under 5 years (adult models only); highest education of any household member; household poverty; urban location; insurance coverage; respondent age, marital status, education, and health status. Access to care models are for populations with need for care.

# Indicates that odds ratios among poor and non-poor differ significantly (p<0.05).

### Access to prevention, need for and access to care among children

Rates of immunization and Vitamin A coverage varied greatly across countries; of concern, in half of countries, only 50% or less of children under 5 years old received at least one measles and DPT vaccine or Vitamin A prophylaxis (Table 3). In most countries, Vitamin A coverage, immunizations, access to care when needed, and malaria treatment did not differ for girls and boys in unadjusted (Figure 4) or multivariate (Table 6) analyses. In multivariate analyses, girls were less likely to have reported illness than boys (Table 6).

**Table 6:** Relationship of gender to prevention and need for and access to curative care among children <5 years old*.

Prevention, need for and access to care	Odds ratio, all households (95% confidence interval)	Odds ratio, poor households (95% confidence interval)	Odds ratio, less poor households (95% confidence interval)
At least one Vitamin A capsule in past 12 months	1.03 (1.00, 1.07)	1.04 (0.99, 1.09)	1.03 (0.98, 1.07)
Measles vaccine received	1.00 (0.98, 1.03)	1.01 (0.96, 1.05)	1.00 (0.96, 1.04)
At least one measles and one DPT vaccine received	1.01 (0.98, 1.04)	1.01 (0.96, 1.05)	1.01 (0.97, 1.05)
All three DPT vaccines received	1.02 (0.99, 1.05)	1.02 (0.97, 1.06)	1.02 (0.98, 1.06)
Fever, severe diarrhoea, or other illness	0.93 (0.90, 0.97)	0.96 (0.92, 1.01)	0.91 (0.87, 0.95)
Malaria episode in past 12 months	1.05 (0.99, 1.12)	1.00 (0.91, 1.09)	1.09 (1.01, 1.18)
Care received for last illness	0.98 (0.94, 1.03)	1.01 (0.95, 1.07)	0.96 (0.9, 1.03)
Care received within 24 hours	1.00 (0.96, 1.04)	0.95 (0.90, 1.01)	1.03 (0.98, 1.09)
Care received in hospital	0.97 (0.94, 1.02)	0.94 (0.88, 1.00)	1.00 (0.95, 1.05)
Care received in public facility	1.05 (0.99, 1.11)	0.98 (0.88, 1.09)	1.08 (1.01, 1.17)
Treatment for malaria during last episode of fever	1.01 (0.96, 1.06)	0.97 (0.89, 1.05)	1.05 (0.98, 1.13)
Anti-malarial prescribed by professional for malaria	0.92 (0.75, 1.13)	0.79 (0.58, 1.09)	1.00 (0.76, 1.32)

* Females are coded as 1 in the models. Models control for household size; having a member age 60 years and older; highest education of any household member; household poverty; urban location; insurance coverage; adult respondent age, marital status, education, and health status. Access to care models are for populations with need for care.

### Gender and poverty

For all child and most adult care variables studied, the effect of gender did not differ significantly by household poverty (Tables 3a and 3c). For the few outcomes where gender – poverty interactions were significant (difficulty with daily activities; diagnosis of depression; diagnosis of diabetes; treatment for chronic conditions; and treatment for angina), there was no clear pattern of gender differences among poor versus non-poor households. Women in poor households were more likely than men to report difficulty with daily activities and depression; this difference was lower in non-poor households. Women in poor households reported more diabetes diagnoses and marginally more treatment for all chronic conditions, while there were no significant differences in less poor households. Women in non-poor households reported lower rates of angina treatment, while there were no significant differences between women and men in poor households (Table 4).

## Discussion

In light of well-known gender gaps in health, education, economic participation and opportunity, and political empowerment [25], some have expressed concerns that women may also have less access to health care and medicines than men [6], [15].

Based on a standardized survey of large populations of adults and children from 53 countries in six regions, our results suggest that women in many countries report significantly more need for health care and medicines than men. However, contrary to expectations, we did not find consistent differences across countries in indicators of access to treatment between women and men needing care. Similarly, there were few significant differences in selected indicators of access to preventive or curative care for children. In addition, gender and care relationships among poor households were similar to those among non-poor households. Thus, although situations in individual countries [26] will likely differ, our aggregated analyses do not support the notion of uniform gender inequity in access to health care or medicines for the selected conditions measured in the WHS.

How can we explain the discrepancy between our findings and prior expectations of gender inequities disadvantaging women? First, adult respondents to the WHS were a heterogeneous group both within and across countries in terms of health needs, access to care, and their determinants; thus, noise could have masked statistical relationships between gender and access to care. Further, relatively few respondents reported chronic conditions, which may in part be due to self-reported ascertainment of these conditions in the WHS. Given self-reported need for chronic care, relatively few respondents reported having access to care. Similarly, in some countries, larger than expected proportions of children under 5 years of age were reported to not have been sick. Thus, samples could have been too small to detect significant gender differences. However, given the overall large WHS sample sizes and number of countries, we would expect to see consistent trends emerge to support evidence of gender inequity, either overall or among the poor; yet our analyses do not show such trends. In addition, we cannot exclude differential reporting of symptoms, diagnoses, or treatments between men and women; given the heterogeneity of cultures represented in the survey, such differences may have biased our results toward the Null.

We did not examine areas of widely-reported health disadvantages for women, such as physical and sexual violence, sexually transmitted diseases, HIV/AIDS, or pregnancy and child birth and cannot comment on possible inequity in access to care for these health needs or on the social factors that might increase women's health risks [27].

Our findings that women report equal or greater prevalence of common health problems and equal access to needed care is consistent with evidence from a number of recent studies, including a report on management of diabetes and cardiovascular risk factors in seven countries [28]; a study of prescribing patterns for men and women with diabetes in Bahrain [29]; one that demonstrated higher age-adjusted prevalence of medicines use among women in Spain [30]; reports of more frequent and earlier access to anti-retroviral treatment among women in countries in Africa, Latin America, and Asia [12], [31], [32]; a UNICEF report [33] showing that treatment for childhood pneumonia, diarrhoea, and malaria does not vary by gender, and studies showing equal access to care for boys and girls in Bangladesh and Tanzania [34], [35].

Several factors may increase rates of diagnoses and access to care among women. Women have more frequent interactions with the health care system, both because of reproductive health needs and because they serve as family caregivers. They may thus have more opportunity for diagnosis and treatment of the types of acute and chronic conditions we studied. In addition, female community health care workers may facilitate access to care for women [36] and men may be more reluctant to seek care for cultural and other reasons [37], [38].

Importantly, the low reported levels of chronic and preventive care for both women and men are disconcerting. For example, in 10 of 52 countries with sufficient data, less than a third of adults with diagnosed diabetes reported treatment. With the prevalence of chronic conditions increasing, health system interventions to enable affordable access to long-term therapy are urgently needed [39]. In 13 of 39 countries, less than a third of children had received Vitamin A prophylaxis in the past year and in 20 of 50 countries, less than a third had received measles and or one DPT vaccine. These results attest to the global need for effective health system interventions to improve child survival [8].

From these data, we cannot know whether women and men utilize the same providers or receive the same quality of health care. Gender-stratified assessment of the management of chronic conditions in seven countries seemed to indicate more ineffective management of blood glucose, blood pressure, and hypercholesterolemia among women with diabetes in four low and middle income countries [28]; others found no gender difference in quality of malaria case management [40].

We also do not know whether women and men faced similar circumstances when negotiating access to care within households and health care systems, or experienced the same economic consequences of accessing care. Given that women generally have less power and are poorer than men [41], they may have to expend more effort to access care and experience greater economic repercussions. However, notably, women reported more satisfaction than men with their health care systems and did not feel that they were treated worse than men because of their gender.

The WHO has called for increased attention to gender and poverty in health research [42]. Data on gender are typically available in facility-level and community-level studies of medicines access and use [43], but gender differences have rarely been the focus of empirical analyses. Reporting results of such studies by gender would provide evidence about the generalizability of these finding in different settings, for other health problems, and over time.

More empirical studies are needed to provide evidence about the interrelationships of gender, poverty, and access to health care and medicines, while social and behavioural studies are needed to understand reasons for differences where they exist, negative impacts on health, and potential mechanisms to redress them. These interrelationships are likely to differ by culture, geography, health system structure, and extent of social protection. Given the dearth of empirical literature, we need studies in a wider variety of settings to begin to disentangle the distinct roles of poverty and gender in determining perceptions of illness, patterns of care seeking, access to services, quality of the care, clinical effectiveness, economic impacts, and consumer satisfaction. Documenting specific gender differences in medicines access, use, and affordability would highlight key policy issues and potential solutions to achieve gender equity.

## Supporting Information

Table S1 **Arthritis diagnosis and treatment.**
(XLSX)

Table S2 **Angina diagnosis and treatment.**
(XLSX)

Table S3 **Asthma diagnosis and treatment.**
(XLSX)

Table S4 **Depression diagnosis and treatment.**
(XLSX)

Table S5 **Schizophrenia diagnosis and treatment.**
(XLSX)

Table S6 **Diabetes diagnosis and treatment.**
(XLSX)

Table S7 **Need for and access to care among adults.**
(XLSX)

Table S8 **Need for and access to care among children.**
(XLSX)
